# Dyslipidaemia for patients with low-energy femoral neck fractures after the treatment of cancellous screws: a retrospective study with a 3-year minimum follow-up

**DOI:** 10.1186/s12891-017-1804-x

**Published:** 2017-11-10

**Authors:** Chi Zhang, Xiaoxiao Zhu, Genwang Pei, Ping Xu, Xianshang Zeng, Lili Zhang, Nan Zhang, Dan Zeng, Lei Cao, Weiguang Yu, Xinchao Zhang

**Affiliations:** 1 The first clinical college of Jinan University, Huangpu Avenue West No.613, Tianhe District, Guangzhou, 510630 China; 2 Department of joint surgery, The Third Affiliated Hospital of Guangzhou Medical University, Duobao Road No.63, Liwan District, Guangzhou, Guangdong 510150 China; 3 Endocrine Department, The First Affiliated Hospital of Sun Yat-sen University, Huangpu East Road No. 183, Huangpu District, Guangzhou, 510700 China; 4 Department of ENT, The First Affiliated Hospital of Sun Yat-sen University, Huangpu East Road No. 183, Huangpu District, Guangzhou, 510700 China; 5 Radiology Department, The First Affiliated Hospital of Sun Yat-sen University, Huangpu East Road No. 183, Huangpu District, Guangzhou, 510700 China; 6 Department of Orthopedics, The First Affiliated Hospital of Sun Yat-sen University, Huangpu East Road No. 183, Huangpu District, Guangzhou, 510700 China; 7 Department of Anesthesiology, The First Affiliated Hospital of Sun Yat-sen University, Huangpu East Road No. 183, Huangpu District, Guangzhou, 510700 China; 8 Ultrasonography Department, The First Affiliated Hospital of Sun Yat-sen University, Huangpu East Road No. 183, Huangpu District, Guangzhou, 510700 China; 9 Department of Anesthesiology, The Central Hospital of Wuhan, Tongji Medical College, Huazhong University of Science and Technology, Gusao Road No. 16, Jianghan District, Wuhan, Hubei 430014 China; 10 Department of Orthopaedics, Jinshan Hospital, Fudan University, Longhang Road No. 1508, Jinshan District, Shanghai City, 201508 China

**Keywords:** Avascular necrosis of the femoral head, High density lipoprotein, Low density lipoprotein

## Abstract

**Background:**

Avascular necrosis of the femoral head (AVNFH) occurs infrequently following femoral neck fracture. The association between AVNFH and dyslipidaemia remains controversial. Although major risk factors for AVNFH have been proposed, most of them remain under discussion. Our purpose herein was to evaluate the association between dyslipidaemia and AVNFH following low-energy femoral neck fractures treated with cancellous screws in elderly patients in our tertiary care centre.

**Methods:**

Four hundred and seventy-two consecutive patients (472 hips) with low-energy femoral neck fractures were identified and treated with cancellous screws from July 2007 to April 2013. Patients underwent evaluations preoperatively and each subsequent postoperative visit (months 1, 6, 12, 18, 24, 30, and 36). Clinical and radiographic evaluations were documented at each visit. The risk factors of AVNFH were assessed by multivariate binary logistic analysis.

**Results:**

Follow-up was available for 277 patients, which included 135 patients diagnosed with AVNFH (AVNFH group) and 142 patients without AVNFH (control group). The median follow-up for patients alive at the time of analysis was 40 months (range, 37 to 46 months). The mean total cholesterol (TC), triglyceride (TG), low density lipoprotein cholesterol (LDL-C), and apolipoprotein B (Apo-B) values were considerably higher in the AVNFH group compared with those in the control group. The mean high density lipoprotein cholesterol (HDL-C) and apolipoprotein A1(Apo-A1) values were significantly lower in the AVNFH group compared with those in the control group. A multivariate logistic backward regression model showed that HDL-C and LDL-C were the only variables associated with the development of postoperative AVNFH in patients with a femoral neck fracture (Odds ratio[OR] 33.09, 95% Confidence Interval[CI]: 2.65–19.42, *p* < 0.001 and OR 45.94, 95% CI: 0.47–27.75, p < 0.001, respectively).

**Conclusion:**

Our results suggest that both low HDL-C and high LDL-C have a tendency to result in the occurrence of AVNFH in elderly patients with low-energy femoral neck fractures treated with cancellous screws.

## Background

Avascular necrosis of the femoral head (AVNFH) is an uncommon but serious disease resulting from a variety of potential factors that can lead to vascular damage [1–3]. Despite a rising incidence of AVNFH, its disease-related causes have not been well documented [1, 4–6]. At present, it is clear that when a fracture occurs the incidence of AVNFH is largely determined by the amount of damage to the blood supply of the femoral head [4]. However, the aetiology of AVNFH and the association between AVNFH and dyslipidaemia remain controversial [7]. Prior studies have suggested that the formation of fat embolisms caused by hyperlipidaemia has played a role in the occurrence of AVNFH [8, 9]; however, this proposal is also controversial [3]. Furthermore, whether or not dyslipidaemia is associated with the occurrence of AVNFH following a femoral neck fracture treated with cancellous screws has rarely been reported in the published literature [6, 8, 10].

The aim of this retrospective study was to evaluate the association between dyslipidaemia and AVNFH diagnosed following femoral neck fractures fixed with cancellous screws in elderly patients.

## Methods

### Settings and study population

This study was approved by the institutional review board of Sun Yat-sen University, which waived the requirement for informed consent. Evidence of the institutional review board approval is shown in the appendix. The listed authors had sole responsibility for the study design, data collection, decision to submit the manuscript for publication, and drafting of the manuscript. After institutional review board approval, we identified 472 patients from our institution with acute femoral neck fractures consecutively treated with closed reduction and internal fixation with cancellous screws (Smith & Nephew, Memphis, Tennessee) from July 2007 to April 2013. This study was designed as a single-institution retrospective study. The orthopaedist who examined a patient in the clinic also performed the surgery. The Garden classification was used to assess fractures. The orthopaedist collected the patient-related data from electronic patient records, a structured patient survey, and a structured electronic form on which the orthopaedist recorded data during the operation. Patients diagnosed with AVNFH were assigned to one group (AVNFH group) for comparison to patients who were not diagnosed as having AVNFH (control group). To identify significant factors associated with AVNFH diagnosed after surgical treatment for femoral neck fractures, univariate analysis combined with multivariate binary logistic analysis was performed. Patients underwent similar surgical techniques with similar operating equipment. Follow-up occurred at 1, 6, 12, 18, 24, 30, and 36 months after surgery. The clinical data and radiographic information were evaluated at each visit.

### Inclusion criteria

The inclusion criteria included patients with initial fractures who underwent treatment for an isolated acute closed femoral neck fracture using cancellous screws from July 2007 to April 2013, were 50–70 years old and completed a minimum of a 3-year follow-up period. All femoral neck fracture types were included (i.e., Garden classification I –IV).

### Exclusion criteria

The exclusion criteria included alcohol abuse, multiple injuries, metabolic abnormalities, developmental dysplasia of the hip (DDH), bed-ridden status, and an American Society of Anesthesiologists (ASA) score of V. To minimize possible outliers, the data from patients who were in other treatment groups during the period of enrolment were excluded from the analysis.

### Definitions of the descriptive variables

One orthopaedist (WY) used the International Classification of Diseases code to diagnose AVNFH based on image data that presented changes in radionuclide imaging or femoral density. Based on this classification, patients were assigned to either the AVNFH group or the control group (no AVNFH). Age was recorded in full years at the time of the surgery. The time between the clinical examination and surgery was recorded in days. Patients’ body mass index (BMI) was calculated during the clinical examination. Bone mineral density (BMD) T-score was measured at the femoral neck. Mechanical failure was defined as the need for additional surgical intervention to replace the internal fixation.

### Blood sample processing and detection variables

Blood was drawn without stasis by clean venipuncture and collected in vacuum tubes containing 75 mmol/L trisodium citrate (DiYou Electronics and Technology Co., Ltd., Shanghai, China) as an anticoagulant. The following blood lipid levels were considered to be AVNFH risk factors: triglyceride (TG), total cholesterol (TC), low density lipoprotein cholesterol (LDL-C), high density lipoprotein cholesterol (HDL-C), apolipoprotein A1 (Apo-A1), and apolipoprotein B (Apo-B). Enzymatic methods were used to determine the concentrations of the variables. Serum LDL-C, the primary efficacy variable, was calculated using the Friedewald equation.

### Statistical analysis

The descriptive and outcome variables were assessed by calculating frequencies, means, and standard deviations (SDs). Comparison between groups was conducted using Pearson’s Chi-square test (Fisher’s exact test was used when necessary) or the Mann–Whitney U test for continuous variables. Multivariate binary logistic analysis was performed to evaluate the association between dyslipidaemia and AVNFH. The coefficients obtained from logistic regression were expressed in terms of odds ratio with 95% confidence intervals. Statistical differences were considered significant for *p* values <0.05. The statistical analysis was conducted using dedicated software (IBM-SPSS Statistics version 23.0, Inc., New York, USA).

## Results

### Comparison of patient and treatment characteristics

**Fig. 1:**
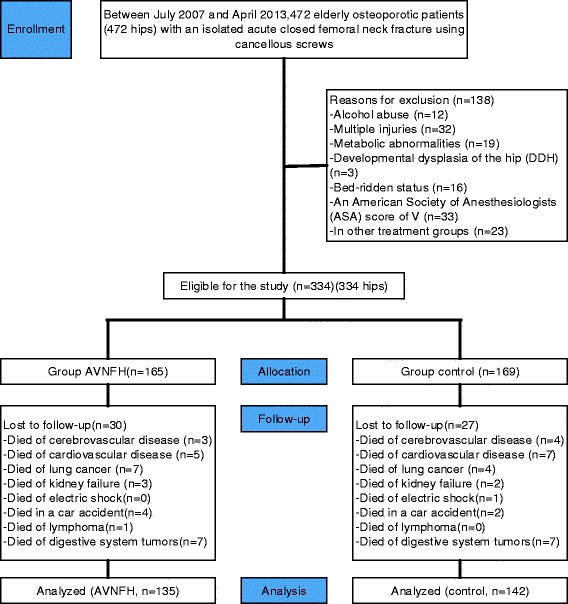
Flow diagram demonstrating methods for identification of studies to evaluate the association between dyslipidaemia and AVNFH following low-energy femoral neck fractures treated with cancellous screws in elderly patients, and reasons for exclusion

Two hundred and seventy-seven patients (AVNFH group *n* = 135; control group *n* = 142) met the study eligibility criteria. Details are shown in Fig. 1. The AVNFH and control groups of patients for each operating surgeon differed slightly regarding their age (70.6 ± 11.71 and 68.4 ± 10.39 years, respectively) and BMI (24.6 ± 1.60 and 24.3 ± 1.74 kg/m^2^, respectively). The mean follow-up was 3.4 years (range, 3–4 years). Table 1 shows that the variability of descriptive characteristics was not statistically significant between the two groups (*p* > 0.05).

**Table 1:** Patient demographics between groups

Variable	AVNFH (n = 135)	Control (*n* = 142)	*P* - value
Age (years)	70.6 ± 11.71	68.4 ± 10.39	0.11^*a^
Sex (M:F)	63:72	68:74	0.84^*b^
ASA scale, No.			0.99^*c^
I	26	29	
II	37	41	
III	53	48	
IV	19	24	
V	0	0	
Laterality (L/R)	84/51	88/54	0.97^*b^
BMI (kg/m^2^)	24.6 ± 1.60	24.3 ± 1.74	0.14^*a^
BMD	2.0 ± 0.41	2.1 ± 0.54	0.11^*a^
Garden classification (No.)	84		0.97^*c^
I	17	20	
II	31	37	
III	44	45	
IV	43	40	

^*^Not statistically significant. ^a^Analysed using an Independent-Samples t-test; ^b^Analysed using the Chi-square test; ^c^Analysed using the Mann-Whitney test. *AVNFH* Avascular necrosis of the femoral head, *ASA* American Society of Anesthesiologists, *BMI* Body mass index, *BMD* Bone mineral density

The statistical significance results of the changes in the treatment outcome variables between groups are summarized in Table 2. Non-significant differences existed between groups on the basis of similar treatment procedures. For 135 patients diagnosed with AVNFH, the median follow-up time from diagnosis was 23 months (range 12–37), but 95/135 cases (70.3%) were identified in a 25-month period after surgery. We identified 37 total cases (13.4%) of mechanical failure during the study period. A total of 24/135 (17.8%) AVNFH patients with mechanical failure underwent a reoperation procedure after a mean of 29 months, which was higher than the control group (8.9%).

**Table 2:** Comparison of the treatment of patients with femoral neck fractures between groups

Variable	AVNFH (n = 135)	Control (n = 142)	*P* - value
HHS	76.9 ± 6.65	79.2 ± 10.0	0.02^*a^
Garden classification			0.73^*b^
I	30	35	
II	28	37	
III	53	43	
IV	24	27	
Injury operation interval			0.93^*b^
< 24 h	21	25	
24–48 h	39	48	
48–72 h	41	32	
> 72 h	34	37	
Weight-bearing activity time (<8 months/≥8 months)	46/89	50/92	0.84^*c^
Mechanical failure	17.8% (24/135)	8.9% (13/142)	0.04^c^

^*^Not statistically significant. ^a^Analysed using an Independent-Samples t-test; ^b^Analysed using the Mann-Whitney test; ^c^Analysed using the Chi-square test. *AVNFH* Avascular necrosis of the femoral head, *HHS* Harris Hip Score

### Comparisons of the average blood lipid levels

A comparison of preoperative mean blood lipid levels between groups is presented in Table 3, but no significant differences were observed. However, significant differences were noted for all variables in the postoperative comparison (*p*-values = 0.00), which is shown in Table 4. Differences in the HDL-C and LDL-C levels in the AVNFH group versus the control group were 1.34 mmol/L (SD 0.43) vs 2.00 mmol/L (SD 0.45) and 3.92 mmol/L (SD 0.64) vs 1.79 mmol/L (SD 0.24), respectively. In the AVNFH group, the TG and Apo-B levels were 2.17 ± 0.30 mmol/L and 79.41 ± 7.32 mg/dl, respectively, which were considerably higher than those of the control group. In contrast, HDL-C and Apo-A1 levels in the AVNFH group were 1.34 ± 0.43 mmol/L and 108.08 ± 5.96 mg/dl, respectively, which were considerably lower than those of the control group. Low HDL-C and high LDL-C were present in 7% and 18% of patients with AVNFH risk factors, respectively.

**Table 3:** Preoperative comparison of average blood lipid levels between groups

Variable	AVNFH (n = 135)	Control(n = 142)	*P* - value
TC (mmol/L)	4.60 ± 0.87	4.42 ± 0.98	0.12^*a^
TG (mmol/L)	1.71 ± 0.45	1.65 ± 0.46	0.24^*a^
HDL-C (mmol/L)	2.65 ± 0.35	2.72 ± 0.50	0.16^*a^
LDL-C (mmol/L)	3.24 ± 0.14	3.20 ± 0.32	0.16^*a^
Apo-A1 (mg/dl)	99.40 ± 11.25	101.04 ± 10.02	0.20^*a^
Apo-B (mg/dl)	59.36 ± 9.11	57.51 ± 10.53	0.12^*a^

^*^Not statistically significant. ^a^Analysed using an Independent-Samples t-test. *AVNFH* Avascular necrosis of the femoral head, *TC* Total cholesterol, *TG* Triglyceride, *HDL* High density lipoprotein, *LDL* Low density lipoprotein, *Apo-A1* Apolipoprotein A1, *Apo-B* Apolipoprotein B

**Table 4:** Comparison of average blood lipid levels between groups

Variable	AVNFH (n = 135)	Control (n = 142)	*P* – value
TC (mmol/L)	6.46 ± 0.89	4.48 ± 0.88	0.00^*a^
TG (mmol/L)	2.17 ± 0.30	1.51 ± 0.25	0.00^*a^
HDL-C (mmol/L)	1.34 ± 0.43	2.00 ± 0.45	0.00^*a^
LDL-C (mmol/L)	3.92 ± 0.64	1.79 ± 0.24	0.00^*a^
Apo-A1 (mg/dl)	108.08 ± 5.96	118.14 ± 9.07	0.00^*a^
Apo-B (mg/dl)	79.41 ± 7.32	76.44 ± 8.82	0.00^*a^

^*^Statistically significant. ^a^Analysed using an Independent-Samples t-test. *AVNFH* Avascular necrosis of the femoral head, *TC* Total cholesterol, *TG* Triglyceride, *HDL-C* High density lipoprotein cholesterol, *LDL-C* Low density lipoprotein cholesterol, *Apo-A1* Apolipoprotein A1, *Apo-B* Apolipoprotein *B*

### Multivariate binary logistic analysis of AVNFH

A multivariate logistic backward regression model was conducted using the previously described blood lipid levels associated with the postoperative occurrence of AVNFH. In the final model, HDL-C and LDL-C were the only variables associated with the development of postoperative AVNFH in patients with a femoral neck fracture (OR 33.09, 95% CI: 2.65~19.42, *p* < 0.001 and OR 45.94, 95% CI: 0.47~27.75, *p* < 0.001, respectively), which is shown in Table 5. Concerning blood lipid parameters, patients with AVNFH had a significantly greater occurrence of low HDL-C and high LDL-C levels than patients without AVNFH (34% vs 14%, *p* < 0.001), respectively. Patients who developed AVNFH during follow-up had a mean LDL-C level (3.92 ± 0.64 mmol/L) significantly higher than that of patients without AVNFH (1.79 ± 0.24 mmol/L) (*p* < 0.001).

**Table 5:** Multivariate binary logistic analysis of factors associated with AVNFH following femoral neck fractures

Influence factors	β	SE	OR	95% CI	χ^2^	*P* - value
TC	0.483	0.442	2.42	1.02~3.05	4.11	0.203
TG	2.717	0.653	7.88	1.22~6.67	27.33	0.194
HDL-C	1.013	0.215	33.09	2.65~19.42	7.69	0.003^*^
LDL-C	0.292	0.439	45.94	0.47~27.75	4.86	0.004^*^
Apo-A1	0.758	1.22	5.15	1.36~9.71	3.54	0.124
Apo-B	1.119	2.015	4.09	1.62~5.47	11.29	0.331

^*^Statistically significant. *TC* Total cholesterol, *TG* Triglyceride, *HDL-C* High density lipoprotein cholesterol, *LDL-C* Low density lipoprotein cholesterol, *Apo-A1* Apolipoprotein A1, *Apo-B* Apolipoprotein B, *SE* Standard error, *OR* Odds ratio, *CI* confidence interval

## Discussion

The current study shows that both high LDL-C and low HDL-C levels are independent risk factors for AVNFH. Another interesting finding of our study is that an increase or decrease in only one risk factor has little effect on outcome. Our results are mostly in agreement with findings in the previous literature [1, 7, 11, 12]. The main factors associated with AVNFH involve the type of fracture, the degree of initial displacement, and age at the time of injury. The occurrence of AVNFH is considered to be directly related to the initial fracture displacement [5, 10, 13]. Conversely, our study indicated that postoperative AVNFH occurred in elderly patients with nondisplaced or minimally displaced neck fractures, meaning that in addition to fractures and surgical techniques there remain other factors, such as dyslipidaemia, that can lead to the occurrence of AVNFH. Our study is the first report evaluating the association between dyslipidaemia and AVNFH following low-energy femoral neck fractures treated with cancellous screws in elderly patients in our tertiary care centre. Additionally, the current results regarding the combination of HDL-C and LDL-C lipid abnormalities are novel. Our findings emphasize the importance of monitoring lipid levels for predicting the occurrence of AVNFH. A proven preventive effect of HDL-C on AVNFH occurrence could improve the functional status and quality of life for elderly patients with femoral neck fractures.

Previous studies of femoral neck fractures have tended to ignore lipid levels among elderly patients, focusing instead on the treatment of fractures [14–18]. Recent evidence indicates that lipid-lowering treatment has beneficial effects on the immune and skeletal systems and on the primary and secondary prevention of coronary heart disease (CHD) [19, 20], and more recent studies have reported that hyperlipidaemia may be one of the most important contributors to postoperative AVNFH [15, 21, 22]. The authors of a French study of 655 patients diagnosed with AVNFH reported that elderly patients with femoral neck fractures who received a postoperative lipid-lowering treatment experienced a significantly lower incidence of AVNFH during the fracture treatment initiation and maintenance periods than patients who received a null lipid-lowering treatment [23]. Ai et al. [24] reviewed 125 medical records from a primary care practice and found that low HDL-C was present in 34% of patients with AVNFH risk factors, which is higher than what we described in the current study (18% in patients with AVNFH). The current findings also show that patients with high LDL-C levels have a noticeably higher incidence of AVNFH after treatment of femoral neck fractures than patients with low LDL-C levels. Multivariate binary logistic analysis exhibited that both low HDL-C and high LDL-C levels were independent risk factors for AVNFH. The results of a study by Sener et al. [18] are in line with our findings that HDL-C and LDL-C could be regarded as independent predictive factors for AVNFH.

There remains a dispute about the pathogenesis of AVNFH induced by lipid metabolism disorders [25]. Preclinical studies have employed a variety of animal models to better understand whether hyperlipidaemia accelerates vascular ageing and decreases BMD [26, 27]. Disturbance of fat metabolism is a manifestation of AVNFH. Hyperlipidaemia persists throughout the course of AVNFH (fatty degeneration and necrosis of bone cells is consistent in most reports) [6]. Wen et al. [27] performed a rabbit model study using an optical microscope, which showed that red marrow decreased significantly and the marrow cavity filled with numerous fat cells. These results suggest that hyperlipidaemia leads to marrow fat formation in the femoral head. Ryoo et al. performed a study of primary bone marrow stromal cells in mice and found that LDL-C could generate the expression of an adipose specific gene (aP2 mRNA) in the stromal cells and that the expression of type I collagen mRNA was simultaneously decreased. In this manner, bone marrow stromal cells could differentiate into adipocytes and reduce differentiation into osteoblasts. Thus, bone marrow stromal cells in the femoral head could differentiate into numerous adipocytes. In addition, the femoral medullary cavity is a semi-enclosed space and the accumulation of fat in the bone marrow has a tendency to lead to high intraosseous pressure, which can further aggravate the microcirculatory disturbance of the femoral head and cause significant hypoxia, ischaemia, and necrosis in bone cells [1, 6, 11]. At the same time, the reduction of osteogenic differentiation lengthens the bone repair process, which accelerates the occurrence of osteonecrosis of the femoral head [7, 12]. Intraosseous vascular embolization induced by hyperlipidaemia can also lead to bone tissue ischaemia by destroying vascular endothelial cells and creating pro-thrombotic conditions [3, 5]. Notably, high microcirculation pressure triggered by fat accumulation contributes to the occurrence of ischaemia, hypoxia, and metabolic disorders which lead to high microcirculation pressure, ischaemia, and hypoxia, forming a vicious circle and eventually triggering the occurrence of AVNFH [6, 12].

A key limitation of the current study was its retrospective nature. Patient- and surgeon-related confounders may have existed, but both groups were well matched, which allowed us to conclude that results were not associated with the patients’ demographics. Prospective studies assessing the association between dyslipidaemia and AVNFH are needed to understand the role of dyslipidaemia in the development of AVNFH and to highlight any causal links.

## Conclusions

Our study provides strong statistical support for the suspected links between dyslipidaemia and AVNFH. Both high LDL-C and low HDL-C can be recognized as predictive factors for AVNFH. Early intervention in patients with hyperlipidaemia should be performed to prevent or slow the occurrence and progression of AVNFH. This study had several limitations; however, our results seemed to be in accordance with some meta-analyses of RCTs [28–30]. There is a trend that MRI, in conjunction with LDL-C and HDL-C levels, appears to be the preferred predictive tool for AVNFH [21].

## Abbreviations

Apo-A1: Apolipoprotein A1
Apo-B: Apolipoprotein B
ASA: American Society of Anesthesiologists
AVNFH: Avascular necrosis of the femoral head
BMD: Bone mineral density
BMI: Body mass index
CHD: Coronary heart disease
DDH: Developmental dysplasia of the hip
HDL-C: High density lipoprotein cholesterol
LDL-C: Low density lipoprotein cholesterol
TC: Total cholesterol
TG: Triglyceride
